# Broad CTL Response in Early HIV Infection Drives Multiple Concurrent CTL Escapes

**DOI:** 10.1371/journal.pcbi.1004492

**Published:** 2015-10-27

**Authors:** Sivan Leviyang, Vitaly V. Ganusov

**Affiliations:** 1 Department of Mathematics and Statistics, Georgetown University, Washington, DC, United States of America; 2 Department of Microbiology, University of Tennessee, Knoxville, Tennessee, United States of America; ETH Zurich, SWITZERLAND

## Abstract

Recent studies have highlighted the ability of HIV to escape from cytotoxic T lymphocyte (CTL) responses that concurrently target multiple viral epitopes. Yet, the viral dynamics involved in such escape are incompletely understood. Previous analyses have made several strong assumptions regarding HIV escape from CTL responses such as independent or non-concurrent escape from individual CTL responses. Using experimental data from evolution of HIV half genomes in four patients we observe concurrent viral escape from multiple CTL responses during early infection (first 100 days of infection), providing confirmation of a recent result found in a study of one HIV-infected patient. We show that current methods of estimating CTL escape rates, based on the assumption of independent escapes, are biased and perform poorly when CTL escape proceeds concurrently at multiple epitopes. We propose a new method for analyzing longitudinal sequence data to estimate the rate of CTL escape across multiple epitopes; this method involves few parameters and performs well in simulation studies. By applying our novel method to experimental data, we find that concurrent multiple escapes occur at rates between 0.03 and 0.4 day^−1^, a relatively broad range that reflects uncertainty due to sparse sampling and wide ranges of parameter values. However, we show that concurrent escape at rates 0.1–0.2 day^−1^ across multiple epitopes is consistent with our patient datasets.

## Author Summary

Since the early 1990s, cytotoxic T lymphocytes (CTLs) have been known to play an important role in HIV infection with CTLs targeting HIV epitopes and, in turn, HIV escapes arising through mutations in the targeted epitopes. Over the past decade, studies have shown that CTL responses concurrently target multiple HIV epitopes, yet the effect of concurrent responses on HIV dynamics and evolution is not well understood. Through an analysis of patient datasets and a novel statistical method, we show that during early HIV infection concurrent CTL responses drive concurrent HIV escapes at multiple epitopes with significant pressure, suggesting a complex picture in which HIV simultaneously explores multiple mutational pathways to escape from broad and potent CTL response.

## Introduction

During Human Immunodeficiency Virus 1 (simply HIV hereafter) infection, cytotoxic T lymphocyte (CTL) responses play a significant role in shaping viral dynamics and evolution [1]. CTLs, which are activated CD8+ T cells, identify and target HIV-infected cells by recognizing parts of viral proteins called epitopes that are displayed on the surface of infected cells in combination with MHC-I molecules [2]. However, as has been noted since the 1990s [3, 4], the error-prone viral reverse transcriptase generates mutations, resulting in altered proteins, and leading to the loss of recognition of infected cells by CTLs. Such mutations and their rise in frequency, referred to as CTL escape, can occur throughout infection but are especially prominent in the weeks following peak viral load [5–7]. Besides playing an important role in shaping viral dynamics and evolution, CTL escapes are a major problem in the development of an effective vaccine [1, 8–10].

HIV-specific CTL responses first arise about 3 weeks into infection, several days prior to peak viral load, and initially target roughly 3–5 epitopes [9, 11]. Typically, at times near peak viral load, T cell responses to 1–2 of the roughly 3–5 epitopes are dominant as measured through ELISpot or peptide-MHC tetramers, with other responses present at low levels [11, 12]. In the weeks following peak viral load CTL response to other epitopes increase, a phenomenon we refer to as broadening [11–13]. During this time, some CTL responses decline in magnitude, often in association with the rise in frequency of mutations at the targeted epitope, while other CTL responses rise in magnitude [9, 11, 12]. Overall, the first two months of HIV infection are marked by several asynchronous but concurrent CTL responses, reflecting a broad CTL response [11–13].

Viral escape from CTL-mediated killing follows a temporal pattern similar to CTL response, although not all responses elicit an escape and escapes do not always occur in the same order as the CTL responses [13]. The first escape mutations rise to significant frequency 1–3 weeks after peak viral load and are usually restricted to the 1–2 epitopes targeted by the dominant CTL responses around the time of peak viral load. Escape mutations at other epitopes reach significant frequencies later, about 4–6 weeks after peak viral load [12–15].

Previous studies have measured the rate of CTL escape as a means of quantifying the strength of CTL response [12, 15–20]. The escape rate is the difference between the growth rates of the escape variant and wild type populations or, more explicitly, the difference between the slope of the escape variant and wild type ln-VL curves [19, 21], where ln-VL denotes the natural logarithm of the viral load attributed to the respective variants. From a population genetics perspective, the escape rate is the selective advantage of the escape variant relative to the wild type [22]. When escape mutations do not reduce replicative fitness, which we define as the growth rate of the virus in the absence of CTL response, the selective advantage of the escape variant is typically attributed to CTL killing, making the escape rate equal to the rate at which an infected cell is killed by CTLs targeting the given epitope [19, 21, 23]. When escape mutations do reduce replicative fitness, the CTL kill rate is greater than the escape rate. Thus, the escape rate provides a quantitative estimate of the in vivo strength of CTL response.

A common approach for estimating escape rates, introduced in [19, 21, 23] and which we refer to as the logistic model, considers escape dynamics at each epitope separately, a simplification that may miss correlations between escapes at separate epitopes and lead to statistically biased estimates. To apply the logistic model, the CTL escape must be sampled at two timepoints, with the escape rate estimate then applying to the interval of time between the first and second sample. However, in many existing datasets, including those we consider below, sampling is temporally sparse with few sample times within the first 2–3 months of infection. For many escapes the first or second sample time provides no information because the escape has not started or is already complete, and for those escapes that are ongoing at both sample times, escape rate estimates are averages over long period of times and may not reflect the dynamics of CTL escape soon after peak viral load. Previous authors have extended the logistic model to cases in which the CTL escape is not sampled at two time points by artificially setting mutant and wild type frequencies to intermediate values at sample times, leading to estimates that may be biased. Methods that estimate escape rates by considering concurrent escape at multiple epitopes have been developed [24–26], but these methods are computationally complex and depend on highly parameterized models.

In this work we consider the CTL response associated with the viral escapes that occur during the first 2–3 months of infection in four previously analyzed patient datasets from the Center for HIV/AIDS Vaccine Immunology (CHAVI) cohort: CH40, CH58, CH77, and CH256 [12, 13]. We analyze the escapes using single genome sequencing and amplification (SGA) data that provides linkage information between different epitopes. Importantly, we do not consider viral escapes that are first sampled at times past 2 months post symptoms, corresponding to roughly 3 months post infection. As a result, CTL responses associated with delayed escapes or no escape are not part of our analysis.

Pandit and De Boer [27] recently noted concurrent CTL escapes based on haplotype reconstruction using deep sequencing data from one patient. Similarly, we observe concurrent CTL escape across all four patients we consider. Half-genome sequence samples from the four patients reveal common patterns of escape. Multiple variants mutated at different epitopes were sampled concurrently, reflecting concurrent CTL escape through different mutation pathways. Further, samples reveal individual viral variants with mutations at multiple epitopes, reflecting CTL escape through linked epitope mutations. Put together, escape occurred through multiple mutation pathways and linked escape occurred within these different pathways.

Using specific examples and simulations, we show that estimates of escape rates based on the logistic model can lead to bias in the presence of multi-epitope escape, with the direction of bias depending on the structure of the escape. Furthermore, we show that application of the logistic model when CTL escape is sampled at a single time point leads to estimates that are severely downward biased (i.e., are underestimates). Importantly, these biases are unrelated to the presence of replicative fitness costs. Instead, bias associated with the logistic model is due to ignoring concurrent escapes and making strong assumptions regarding mutant frequency when mutants are undetectable.

To address these limitations in the logistic model, we introduce a novel method for estimating escape rates that removes bias associated with multi-epitope escape by applying the logistic model to pairs of variants, thereby generalizing the notion of wild type and mutant to the setting of multiple epitopes. This novel method still suffers from bias when CTL escape is captured at a single time point, so we introduce a further extension, involving the introduction of three parameters *A*, *t*_*I*_, and *μ*, that allows for unbiased estimation of escape rates given sampling of CTL escape at a single timepoint. Parameter *A* denotes the number of mutations that occur during the escape, *t*_*I*_ is the time at which the first escape mutation occurs, and *μ* is the per epitope virus mutation rate. For a given patient, different CTL escapes will be parameterized by different values for *A*, *t*_*I*_ and *μ* that will typically be unknown. The three parameters are the price we pay for requiring only a single sample time during each escape. Through simulation, we show that when *A*, *t*_*I*_ and *μ* are known, our estimates are not biased by multi-epitope response and single time point sampling.

Although we are unable to give a narrow range for the rate at which escapes proceed, our results show that escape rates in the range of 0.1–0.2 day^−1^ across multiple epitopes are consistent with our dataset. Such significant escape rates across multiple epitopes would reflect a broad yet still relatively strong CTL killing. If replicative fitness costs associated with escape exist, CTL kill rates are above the range 0.1–0.2 day^−1^, suggesting even stronger CTL killing. Importantly, the wide range of escape rates contained within our lower and upper bound reflects a range of model assumptions consistent with our datasets. More accurate escape rate estimates require either more model assumptions, corresponding to narrower choices for *A*, *t*_*I*_ and *μ*, or datasets with denser temporal sampling.

## Results

### Patient Escape Graphs

Goonetilleke et. al. [12] and Liu et. al. [13] identified putative epitopes targeted by CTL response in 17 patients of the CHAVI cohort including the four patients that we analyze here. Importantly, for every patient in the CHAVI cohort, most putative epitopes with escape mutations in the first several months post infection elicited an experimentally measured epitope-specific CTL response, providing evidence that escape during acute infection is largely driven by CTL mediated selection and does not arise due to neutral evolution [9, 12].

In each of our four patient datasets, two sample time points fall within the first 2–3 months of infection: we label these sample times *t*_1_ and *t*_2_. To analyze early CTL escape, we consider only putative epitopes with mutation in at least one sequence collected at *t*_1_ and *t*_2_. Further, to avoid variation unassociated with CTL response, we only consider putative epitopes that meet at least one of two criteria: 1) the putative epitope is supported by ELISpot assays in [12, 13] or 2) the putative epitope mutated into multiple haplotypes across the sequences collected at *t*_1_ and *t*_2_ (i.e. different sequences collected at *t*_1_ and *t*_2_ had different mutations on the putative epitope) and the putative epitope was eventually lost as infection progressed [12, 28, 29]. Regarding the second criterion, epitope mutation into multiple haplotypes is a typical pattern of CTL escape, termed epitope shattering in [30], and the eventual loss of a putative epitope supports positive selection rather than neutral evolution.

**Fig 1:**
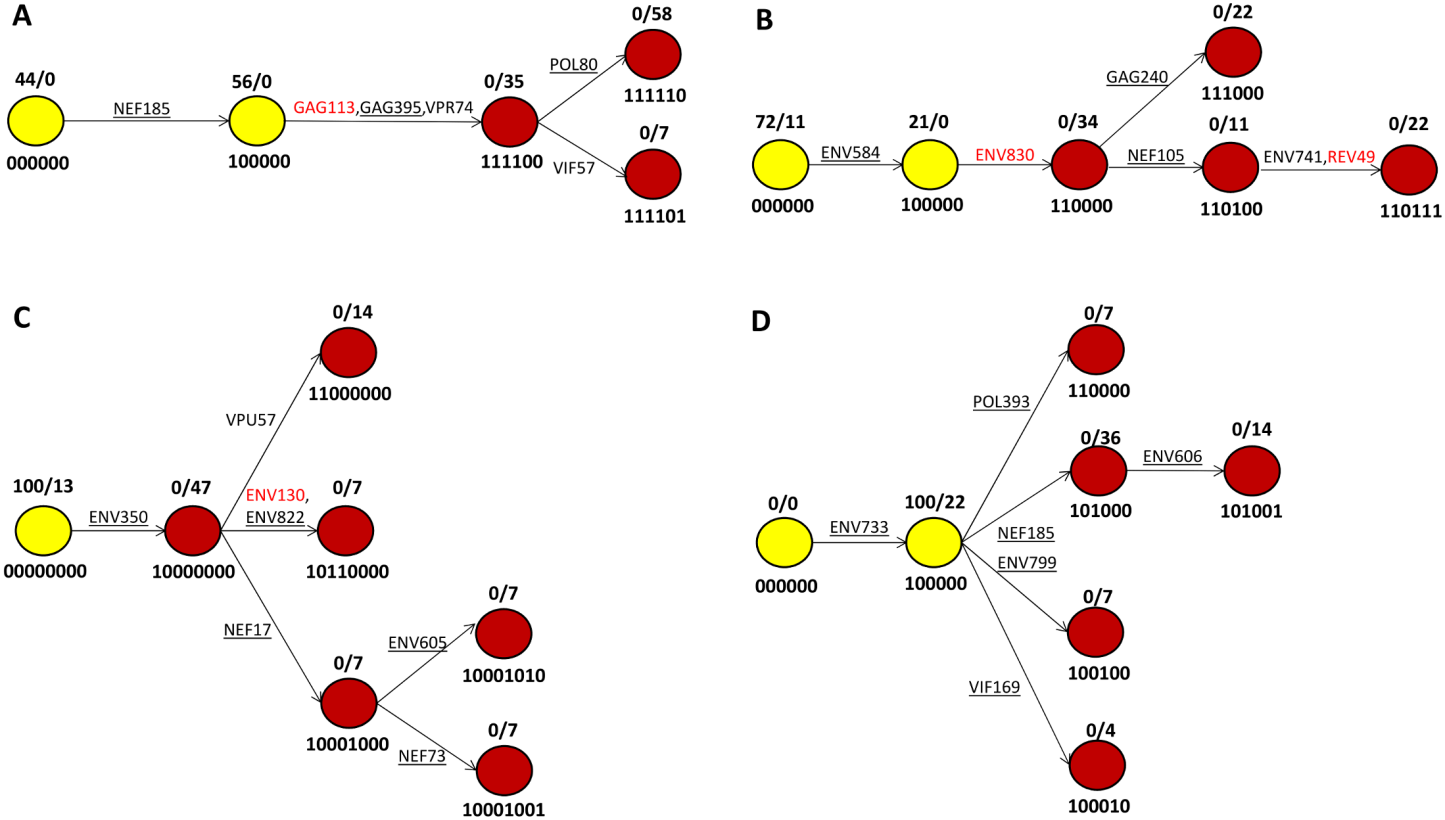
HIV follows a similar escape pattern in multiple patients (panel A: CH40, panel B: CH58, panel C: CH77, panel D: CH256). Vertices of an escape graph represent viral variants that are part of the escape pathway and edges correspond to epitope mutations needed to change one variant into another. Numbering below each vertex indicates whether a particular putative epitope is wild-type (0) or escape (1). Numbers above each vertex that are separated by a slash represent percent of this particular variant at the two time points *t*_1_ and *t*_2_, respectively. For example, in Fig 1A, the vertex numbered below by 111100 corresponds to a viral variant mutated at putative epitopes NEF185, GAG113, GAG395, and VPR74, but not at putative epitopes POL80, VIF57 and comprises 0% and 35% of the sequences sampled at *t*_1_ and *t*_2_, respectively. Yellow and red vertices represent initial variants and expansion variants, respectively; in all patients, initial mutations lead to the first escape which is followed by the expansion of viral variants. CTL responses to putative epitopes are supported by ELISpot assays (underlined epitopes), HLA association (non-underlined, black text epitopes), and multiple mutant haplotypes (red text epitopes). See S3 Table for *t*_1_ and *t*_2_ values and [12, 13] for original descriptions of patient datasets.

Following [24, 25], we model the pathways of CTL escape in each patient through an escape graph. Vertices of an escape graph represent viral variants that are part of the escape pathway and edges correspond to epitope mutations needed to change one variant into another. Variants are characterized by whether the considered epitopes are mutated or not, and variants are labeled by a string of 0’s and 1’s, e.g. 100000, with each digit associated with an epitope and a digit of 0 and 1 representing a haplotype with the epitope un-mutated and mutated, respectively. We do not distinguish between different mutations on the same epitope. Fig 1 shows the escape graphs for patients CH40, CH57, CH77, and CH256, see Methods for details regarding the datasets and construction of escape graphs.

The sample frequencies of each variant in the escape graph at *t*_1_ and *t*_2_ are shown in Fig 1. Over all patients and all variants sampled, only 3 variants are present at both sample times. Given this pattern, for each patient we distinguish between two groups of variants: initial variants and expansion variants. Initial variants are those variants found at the first sample time while expansion variants are found only at the second sample time. Below, we refer to initial or expansion vertices, rather than variants, when we are discussing the escape graph itself rather than the viral population.

The form of the escape graphs reflects the concurrent nature of HIV escape in the four patients and is in-line with the previous results of Pandit and De Boer [27]. For example, as shown in Fig 1D, at *t*_2_ the CH256 escape involves four variants that are all children of variant 100000: 110000, 101000, 100100, and 100010. Escape through these four variants involves mutation at distinct epitopes, reflecting concurrent escape from CTL responses to POL393, NEF185, ENV799, and VIF169 through multiple pathways. For NEF185, escape occurs concurrently through variants 101000 and 101001, with variant 101001 linking escape at NEF185 and ENV606. Similar patterns are seen in the other patients. As shown in Fig 1, most putative epitopes elicited a response in ELISpot assays, providing strong statistical support for escape driven by CTL mediated selection (see S1 Text and S1 Table for further statistical details). Putative epitopes that did not elicit a response in ELISpot assays may not have been exposed to CTL-mediated selection, but our results are unchanged if those are removed from the escape graphs.

At sample times after *t*_2_, a new set of vertices arise from the expansion vertices and the expansion vertices collapse to low frequency or are no longer sampled. For sample times extending up to 6 months, most variants are seen once at intermediate frequencies and then disappear at the next sample time as occurs for the initial vertices in moving from *t*_1_ to *t*_2_. This observation has been made previously, although not in the context of linked data [12, 14, 20, 25].

### Escape Rate Estimates: The Logistic Model

Current methods for estimating escape rates consider escape at each epitope separately by grouping variants into wild type and mutant according to the absence or presence, respectively, of mutation solely at the given epitope [12, 15, 19, 21, 28]. Letting *f*_WT_(*t*) and *f*_MT_(*t*) be the frequency of the wild type and mutant groups at time *t*, the model introduced in [19, 21], which we refer to as the logistic model, leads to an escape rate estimate *ϵ* as the solution of the relation

$$\frac{f_{\text{MT}}\left(t_{2}\right)}{f_{\text{WT}}\left(t_{2}\right)}=\frac{f_{\text{MT}}\left(t_{1}\right)}{f_{\text{WT}}\left(t_{1}\right)}\exp\left[\epsilon \left(t_{2}-t_{1}\right)\right],$$ (1)

with sampling occurring at the two timepoints *t*_1_ and *t*_2_.

Escape rate estimates based on Eq (1) are problematic for three reasons: the presence of multi-epitope escape biases the estimates in unpredictable ways; when wild type or mutants are at zero frequency or unsampled at *t*_1_ and *t*_2_, Eq (1) cannot be applied; and the presence of mutation biases estimates up.

**Fig 2:**
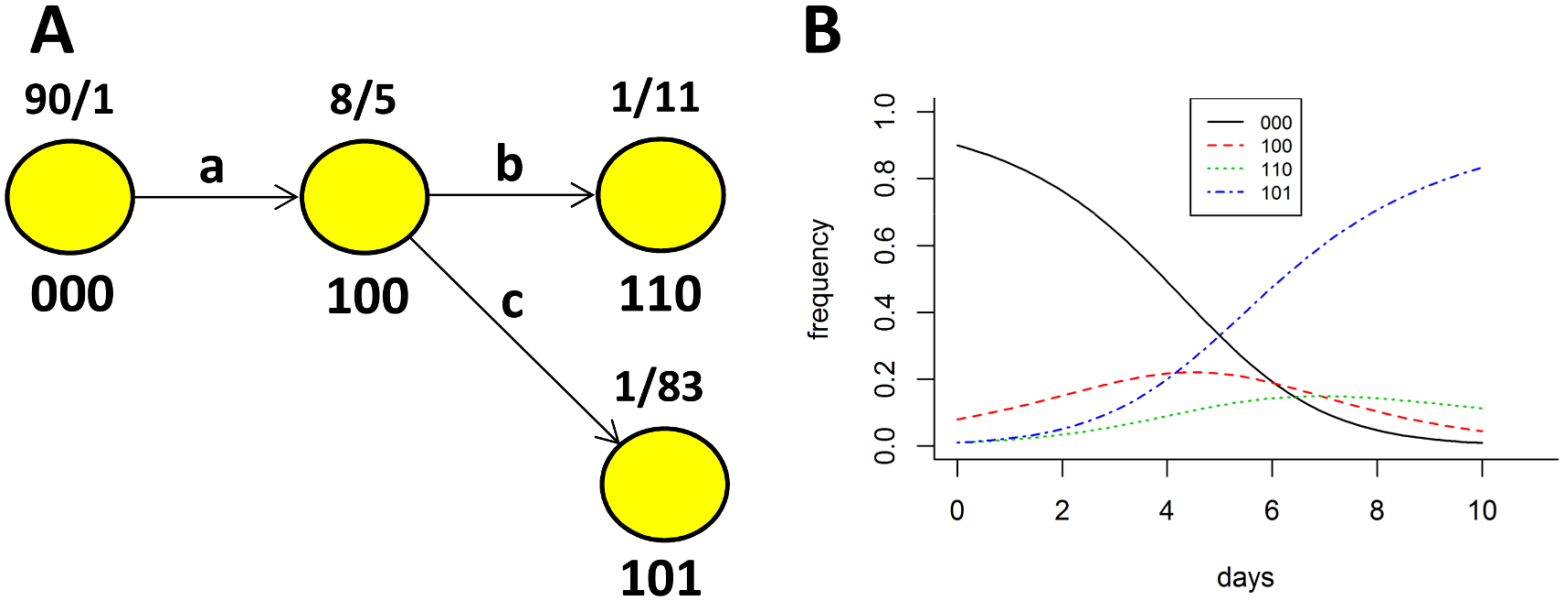
Example of an escape graph generated by sampling the virus population at two time points. We simulate dynamics of viral escape from constant CTL response with killing rates of 0.4, 0.3, and 0.5 day^−1^ against epitopes a, b, and c, respectively. All variants have equal replicative fitness, mutation is ignored, and dynamics are generated deterministically according to Eq (1). Simulation is started at day *t*_1_ = 0 with the initial frequencies shown to the left of the slashes and run to *t*_2_ = 10. In this case *t*_1_ = 0 does not model initial infection; instead it simply serves as the initial time point. The escape graph obtained by sampling the virus population at times *t*_1_ = 0 and *t*_2_ = 10 days with *N* = 15 sequences is shown in panel A and the dynamics of different viral variants are shown in panel B.

In the presence of multi-epitope escape, variants within and across the wild type and mutant groups can differ at epitopes other than the one used to form the groupings, leading to bias. As a concrete example, consider the hypothetical escape graph shown in panel A of Fig 2. The graph reflects escape at epitopes a, b, and c, with the associated frequency dynamics shown in panel B. The frequency dynamics were generated assuming equal replicative fitness across variants and constant CTL kill rates of 0.4, 0.3, and 0.5 day^−1^ at epitopes a, b, and c, respectively. Under these assumptions, the escape rate at each epitope should equal the CTL kill rate at the epitope. However, applying Eq (1) gives escape rate estimates of 0.69, 0.25, and 0.62 day^−1^ for epitopes a, b, and c respectively.

The overestimate of the escape rate at epitope a (0.69 instead of the true 0.4) is caused by the groupings: the wild type group is composed of variant 000 and the mutant group is composed of variants 100, 110, and 101. The escape of mutant variants 110 and 101 is driven by CTL responses to epitopes a, b and c, but is erroneously attributed solely to CTL response to epitope a, leading to the overestimate. Similarly, the underestimate of the escape rate at epitope b (0.25 versus the true value of 0.3) results from a wild type group that includes variant 101 while the mutant group contains variant 110. The escape rate of mutant variant 110 relative to wild type variant 101 is slowed by response at epitope c and Eq (1) erroneously attributes the slower escape to weaker response at epitope b, leading to the underestimate. Overall, different escape graph geometries, variant frequencies, and underlying escape rates can lead to an upward or downward bias in the escape rate estimates given by Eq (1) (see [31] for similar observations).

A second problem associated with Eq (1) arises when either the wild type or mutant groups have sample frequency zero at *t*_1_ or *t*_2_. This occurs when no mutations have occurred by time *t*_1_ or *t*_2_, when the sweep of the mutants has completed and no wild types exist, or when the modest number of samples possible in SGA fails to capture mutant or wild type variants. In such cases Eq (1) cannot be applied: a serious limitation for our patient escape graphs because nearly all epitope mutations are first sampled at *t*_2_ (Fig 1). Previous authors have estimated escape rates in such cases by replacing zero frequencies with a non-zero value, usually 1/(*n* + 1) where *n* is the number of sequences sampled [19–21]. Such an approach leads to lower bounds on the escape rates—in other words a downward bias of the estimates—but as we show below, the lower bounds are poor.

Finally, Eq (1) ignores the presence of mutation. When wild type and mutants are at significant frequencies at *t*_1_ and *t*_2_, mutation plays a minor role. However, in situations when the mutant population is still small at *t*_1_, mutations significantly increase the number of mutants at *t*_2_ relative to *t*_1_ and Eq (1) erroneously attributes these extra mutant variants to a higher escape rate.

Table 1 shows the relative error of escape rate estimates using Eq (1) based on simulations of early acute HIV infection (see caption of Table 1 and Methods for full details of simulations). The simulations include different strengths of CTL response and different escape graph geometries. Column *sampled freq* gives the relative error of the estimate formed through Eq (1) with 15 sampled sequences at both *t*_1_ and *t*_2_. All three biases discussed above are in effect, but the dominant bias is the small frequency of mutants at *t*_1_, leading to samples in which the mutants are not present and the 1/(*n* + 1) substitution is made. The relative errors in this column are all negative, reflecting underestimates of the true escape rates, and extreme: the confidence intervals range between roughly −0.50 and −0.95, which corresponds to escape rate estimates that are 50% to 95% of the true escape rate. To make the effect of these underestimates concrete, consider that a 95% underestimate would give an estimate of 0.03 day^−1^ for a true escape rate of 0.6 day^−1^.

**Table 1:** Conventional methods dramatically underestimate the rate of of viral escape from CTL responses. We ran stochastic simulations of viral escape assuming CTL response at 6 epitopes with an early CTL response at a single epitope followed roughly a week later by CTL response at five additional epitopes. For each simulation, we estimate the escape rate at the 5 additional epitopes using Eq (1) assuming sampling at *t*_1_ = 30 and *t*_2_ = 60 and then calculate the relative error of the estimated escape rate, (estimate-true rate)/true rate, based on sampling *N* = 15 sequences at *t*_1_ and *t*_2_ (column “sampled freq”), based on the exact frequencies of wild type and mutant variants at *t*_1_ and *t*_2_ (column “exact freq”), and based on exact frequencies as well as a model in which no mutations occur after *t*_1_ (column “exact freq/no mutation”). Due to the later CTL response, the 5 additional epitopes correspond to expansion variants. We show relative errors of escape rate estimates under linear and full escape graphs. The linear escape graphs can include only the variants 000000, 100000, 110000, …, 111111. The full escape graphs can include all 2^6^ possible haplotypes formed by wild type and mutants at the 6 epitopes. Strong and weak CTL response reflect simulations in which the killing rate at the 5 additional epitopes had a maximum value of *k* = 0.3 day^−1^ and *k* = 0.12 day^−1^, respectively, with the exact kill rate varying across simulations (see Methods for details). We assume equal replicative fitness across all variants. Confidence intervals (CIs) presented are based on 1,000 simulations for each case of CTL response and escape graph type.

CTL response	graph	sampled freq	exact freq	exact freq/no mutation
strong	linear	-0.65 (-0.89,-0.49)	0.52 (0.31,1.07)	0.37 (0.24,0.87)
weak	linear	-0.96 (-0.98,-0.75)	1.29 (0.82,2.42)	0.85 (0.56,1.49)
strong	full	-0.64 (-0.84,-0.47)	-0.13 (-0.3,0.02)	-0.28 (-0.62,-0.09)
weak	full	-0.95 (-0.97,-0.74)	0.16 (0.02,0.43)	-0.19 (-0.36,-0.07)

Column *exact freq* gives relative errors for estimates formed through Eq (1) using the exact wild type and mutant frequencies and restricted to those escape for which mutants and wild types both existed at times *t*_1_ and *t*_2_, thereby removing the effect of sampling variance and the 1/(*n* + 1) substitution. With the bias associated with the 1/(*n* + 1) substitution removed, only the biases associated with multi-epitope escape and mutation still exist, but these lead to significant under or overestimation of the escape rate depending on the specific form of the simulations.

Finally, to form the column *exact freq/no mutation*, we simulated viral dynamics with no mutation between *t*_1_ and *t*_2_ and then applied Eq (1) using the exact variant frequencies. With the bias of the 1/(*n* + 1) substitution and mutation removed, only the bias associated with multi-epitope escape remains. As can be seen, removing mutation produces lower estimates relative to column *exact freq*, but the estimates are still biased due to multi-epitope escape, with the direction of bias depending on the geometry of the escape graph.

### Escape Rate Estimates: Multi-Epitope Method Using Logistic Model

To account for concurrent multi-epitope escape our approach is to associate an escape rate with each edge of the escape graph. Intuitively, each parent-child vertex pair represents a “competition assay” measuring the selective advantage of the child relative to the parent and since there are only two variants considered, the bias associated with multi-epitope escape in Eq (1) does not occur. Let *f*_P_(*t*) and *f*_C_(*t*) be the parent and child frequencies at time *t* for a given parent-child vertex pair. We generalize the logistic model to parent-child vertex pairs through

$$\frac{f_{\text{C}}\left(t_{2}\right)}{f_{\text{P}}\left(t_{2}\right)}=\frac{f_{\text{C}}\left(t_{1}\right)}{f_{\text{P}}\left(t_{1}\right)}\exp\left[\epsilon \left(t_{2}-t_{1}\right)\right].$$ (2)

In [19, 21], Fernandez et. al. and Asquith et. al. derive Eq (1) by considering the difference in growth rates between wild types and mutants, see for example Equation (1) in [19]. Eq (2) follows from identical arguments, but we consider the parent and child variants instead of wild type and mutant variants. In Eq (1), *f*_WT_(*t*) + *f*_MT_(*t*) = 1 for both *t* = *t*_1_, *t*_2_, but in Eq (2), *f*_P_(*t*) + *f*_C_(*t*) can sum to any value less than or equal to 1.

Eq (2) no longer suffers from multi-epitope escape bias, but the zero frequency and mutation biases still exist. Table 2 is analogous to Table 1, but we estimate escape rates using Eq (2). As column *exact freq/no mutation* in the table shows, estimates are excellent when sampling is exact and no mutation occurs. The errors that do exist in assuming exact frequencies and no mutation arise from numerical error in the simulations and recombination.

**Table 2:** Estimating escape rates using parent-child variants removes the bias associated with concurrent escapes. We use the same simulations as described in Table 1, but estimate escape rates using Eq (2). As in Table 1, we show relative error in estimated escape rates. Note that in this analysis we track escape variant haplotypes using linkage information available from simulations. Notations are identical to those shown in Table 1. Of note, negative bias less than negative one implies that the method estimates negative escape rate (decline in frequency of the escape variant).

CTL response	graph	sampled freq	exact freq	exact freq/no mutation
strong	linear	-0.92 (-1.24,-0.71)	0.03 (0.01,0.07)	-0.01 (-0.02,-0.01)
weak	linear	-1.57 (-1.95,-1.32)	0.16 (0.1,0.3)	-0.04 (-0.06,-0.02)
strong	full	-0.83 (-0.99,-0.68)	0.02 (0,0.05)	-0.01 (-0.03,0.01)
weak	full	-1.49 (-1.84,-1.22)	0.18 (0.11,0.33)	0.01 (-0.01,0.04)

Zero frequencies are particularly problematic in the context of Eq (2) because both parent and child variants can be zero at *t*_1_, while in Eq (1) wild type and mutants cannot both be zero. Substitution of 1/(*n* + 1) for both parent and child frequencies in Eq (2) leads to underestimates that are more extreme than substitution for wild type and mutant frequencies in Eq (1), compare the *sample freq* columns in Tables 1 and 2.

For our patient escape graphs, most vertices are expansion vertices and have zero frequency at *t*_1_. This difficulty exists in our simulations as well, where roughly 90% of all variants have a true frequency of less than 0.1 at *t*_1_ = 30 days, reflecting the time needed for mutant variants to arise and expand to significant frequencies. As a result, Eq (2) is not useful for investigation of early escape rates given sampling times that capture CTL escape at a single time point, as is the case in our patient datasets.

### Escape Rate Estimates: Multi-Epitope Method Using a Mutation Model

To estimate escape rates for edges pointing to expansion vertices, we replace Eq (2) with an estimate that accounts for parent to child mutations and depends on sampled frequencies only at *t*_2_:

$$\frac{f_{\text{C}}\left(t_{2}\right)}{f_{\text{P}}\left(t_{2}\right)}=\frac{A\mu }{\epsilon }\;(\exp[\epsilon \left(t_{2}-t_{I}\right)]-1).$$ (3)

In Eq (3), *A* parameterizes the number of parent to child mutations, *t*_*I*_ is the time of the first parent to child variant mutation, and *μ* is the rate at which a parent variant mutates into a child variant. Given data providing the frequencies *f*_C_(*t*_2_), *f*_P_(*t*_2_) and a choice for the parameters *A*, *t*_*I*_ and *μ*, an estimate *ϵ* is determined through Eq (3). Importantly, Eq (3) estimates escape rates during the interval [*t*_*I*_, *t*_2_] rather than [*t*_1_, *t*_2_]. In practice, since Eq (3) is nonlinear with respect to *ϵ*, a numerical equation solver must be used to calculate *ϵ*.

To explain Eq (3) and the parameters *A*, *t*_*I*_, we consider different models for the ratio *f*_C_(*t*_2_)/*f*_P_(*t*_2_). Let *m*_P_(*t*) and *m*_C_(*t*) be the parent and child variant population sizes at time *t*, respectively. A deterministic model of parent-child dynamics assumes growth rates *r* − ϵ* and *r* for parent and child variants, respectively, and a parent to child mutation rate *μ*, leading to the following differential equation system:

$$\begin{array}{l} {\dot{m}}_{\text{P}}=\left(r-\epsilon^{*}\right)m_{\text{P}},\\ {\dot{m}}_{\text{C}}=rm_{\text{C}}+\mu m_{\text{P}}. \end{array}$$ (4)

In Eq (4), we let ϵ* be the modeled (true) selective advantage of mutants to distinguish it from the escape rate estimate *ϵ* in Eq (3). The selective advantage can arise through CTL killing, replicative fitness costs or other means—the model is unaffected by the underlying cause—and an exact estimate would yield *ϵ* = ϵ*. Integration of Eq (4) from time 0 to *t*_2_ under the initial condition *m*_C_(0) = 0 and *m*_P_(0) = *m*_0_ gives

$$\frac{f_{\text{C}}\left(t_{2}\right)}{f_{\text{P}}\left(t_{2}\right)}\approx \frac{\mu }{\epsilon^{*}}\;(\exp[\epsilon^{*}t_{2}]-1).$$ (5)

Eq (3) is precisely Eq (5) when *A* = 1 and *t*_*I*_ = 0, reflecting the assumptions implicit in Eq (5) that mutation begins at time 0 and occur deterministically with rate *μm*_P_(*t*). While we have presented Eq (4) with constant rate *r* and *μ*, the derivation of Eq (4) would still hold if *r* and/or *μ* vary.

Starting mutation at *t* = 0 is unrealistic, since parent variants do not even exist at initial infection unless the parent is the founder variant, which is not the case for any of the expansion edges in our patient escape graphs. But we can alter Eq (4) to account for delayed mutations as done in [32] by specifying a cutoff time *t*_cutoff_ before which no mutations occur and after which mutations occur at rate *μm*_P_(*t*). Under such a model we would have the relation,

$$\frac{f_{\text{C}}\left(t_{2}\right)}{f_{\text{P}}\left(t_{2}\right)}\approx \frac{\mu }{\epsilon^{*}}\;(\exp[\epsilon^{*}\left(t-t_{\text{cutoff}}\right)]-1),$$ (6)

which is Eq (3) with *A* = 1 and *t*_*I*_ = *t*_cutoff_.

Other models of parent-child variant dynamics and mutation are possible. If we assume that parent variants mutate into child variants at a rate *μm*_P_(*t*) once *t* > *t*_*I*_, but that the actual number of mutations varies around this average due to mutational stochasticity, we arrive at Eq (3) with *A* parameterizing an excess or dearth of mutations relative to the average. When *A* = 1, the number of parent to child mutations exactly equals the average. When *A* > 1 and *A* < 1, the number of mutations is greater and less, respectively, than what we would expect.

Table 3 shows the effect of the parameters *A*, *t*_*I*_, *μ* on escape rate estimates. For the simulations presented in Table 1, we recorded the true *A*, *t*_*I*_ and *μ* value for each expansion edge. The columns of Table 3 show the relative error of escape rate estimates based on Eq (3) when we set one of the parameters as specified in the column label while the other two are set to their true values—confidence intervals (CIs) have not been included for readability, see S2 Table. For example, in the column *A* = 0.75, we set *A* = 0.75 while *t*_*I*_ and *μ* were set to their true values. The true value of *μ* was 10^−4^ for all parent-child pairs. As the table shows, changing any one of the parameters within a reasonable range can lead to estimates that are upper or lower bounds, reflected by a positive or negative relative error, respectively.

**Table 3:** Impact of the model parameters *A*, *μ*, *t*_*I*_ on the estimates of the escape rates. We use the same simulations as described in Table 1 to investigate the impact of *A*, *μ*, *t*_*I*_ on escape rate estimates using Eq (3). We set one of *A*, *μ*, *t*_*I*_ to a value, as shown in the column labels, and set the other two values to the true value of the escape, which we record during the simulations, and then compute the relative error of the escape rate estimate. Changing the value of each of the three parameters within a reasonable range can lead to under or over estimates.

CTL response	graph	*A* = .75	*A* = 10	*t*_*I*_ = 0	*t*_*I*_ = 30	*μ* = 3 × 10^−5^	*μ* = 6 × 10^−4^
strong	linear	0.07	-0.35	-0.38	0.43	0.22	-0.33
weak	linear	0.12	-0.82	-0.43	0.56	0.48	-0.69
strong	full	0.13	-0.28	-0.38	0.39	0.2	-0.29
weak	full	0.18	-0.74	-0.42	0.57	0.44	-0.65

We do not know the ‘true’ *A* or *t*_*I*_ for a parent-child pair within a given patient and *μ* is only roughly known from existing mutation studies [33]. Further, within a given patient different parent-child pairs likely have different ‘true’ *A* and *t*_*I*_ values. Previous approaches for estimating escape rates across multiple epitopes using the standard model and/or birth death processes can be viewed in the context of Eq (3), with the model selected providing an implicit choice for the *A*, *t*_*I*_ and *μ* for each parent-child vertex pair.

To apply Eq (3) to our patient escape graphs, we consider a plausible range for each parameter and develop lower and upper bounds for the escape rates based on these ranges. All escape rates within these lower and upper bounds are consistent with our patient datasets. Conversely, greater certainty in the escape rate estimates requires either more assumptions, i.e. some method of specifying a narrower range for *A*, *t*_*I*_ and *μ*, or denser temporal sampling that would capture escape variants at two timepoints, thereby eliminating expansion variants and allowing us to apply Eq (2).

In our simulations, *A* ∈ [1, 10] and *t*_*I*_ ∈ [16, 34] for roughly 75% of expansion variants. Since peak viral load generally occurs 21 days post infection [9], *t*_*I*_ falls between 5 days prior and two weeks after peak viral load. As an additional verification of the lower bound on *t*_*I*_, the CTL response likely arises roughly a week prior to peak viral load [14], making the presence of an expansion variant that is two or more epitope mutations removed from the wild type unlikely at five days prior to peak viral load. Finally, we take *μ* ∈ [3 × 10^−5^, 3 × 10^−4^]. Given a per nucleotide, per reverse transcription mutation rate of 3 × 10^−5^ [33], the range reflects epitope mutations formed by 1 to 10 different single nucleotide mutations. Applying Eq (3) with *A* = 10, *t*_*I*_ five days prior to peak viral load and *μ* = 3 × 10^−4^ and with *A* = 1, *t*_*I*_ two weeks post peak viral load and *μ* = 3 × 10^−5^, respectively, gives us lower and upper bounds for the escape rate. We also consider intermediate values for our parameters determined by the medians of *A* and *t*_*I*_: *A* = 1.25 and *t*_*I*_ four days post peak viral load. We choose *μ* = 10^−4^ as an intermediate value of *μ*.

Table 4 shows the relative error of these lower and upper bounds applied to the simulations described above, as well as the relative error given the intermediate values of the parameters. Notice that the relative error is negative and positive, respectively, for the lower bound and upper bounds, reflecting under and over estimation of the true escape rate, respectively. The intermediate bounds are roughly accurate, although they tend to slightly overestimate the escape rate.

**Table 4:** Novel method allows for estimates of the escape rate by considering a range of values for *A*, *μ*, *t*_*I*_. We use the same simulations as described in Table 1 and estimate escape rates using Eq (3). Shown are the relative errors of the escape rate estimates. The lower and upper bounds lead to under and over estimates of the escape rate and are calculated using *A* = 10, *t*_*I*_ = −5 days (5 days before peak viral load), *μ* = 3 × 10^−4^ and *A* = 1, *t*_*I*_ = 14 days post peak viral load, *μ* = 3 × 10^−5^, respectively. The intermediate values are calculate using *A* = 1.25, *t*_*I*_ = 4 days post peak viral load, and *μ* = 10^−4^. Lower and upper bounds provide an estimated range for the escape rate, while the intermediate estimate reflects escape rates assuming less extreme parameter choices.

CTL response	graph	lower bound	upper bound	intermediate
strong	linear	-0.56 (-0.84,-0.42)	1.09 (0.95,1.3)	0.14 (0.09,0.2)
weak	linear	-1.27 (-1.57,-1.09)	1.79 (1.51,2.27)	0.13 (0.06,0.22)
strong	full	-0.5 (-0.74,-0.39)	1.01 (0.81,1.21)	0.15 (-0.01,0.22)
weak	full	-1.16 (-1.52,-0.93)	1.83 (1.56,2.47)	0.21 (0.14,0.34)

While the parameter ranges we have chosen are relatively broad, the possibility exists that we have missed the true parameter range of HIV infection. Our method does not eliminate parameter dependence, but by choosing a broad range of parameter values, we are more likely to have captured true dynamics than if we simply specified particular values for *A*, *t*_*I*_ and *μ*.

### Patient Escape Rates

Table 5 provides escape rate estimates for CTL escapes seen in the four patients given the frequencies sampled at *t*_1_ and *t*_2_ (Fig 1). To apply Eq (3), we only calculate escape rates for edges corresponding to a mutation at a single putative epitope and pointing to expansion vertices, but this restriction still includes most putative epitopes in the escape graphs. We calculate escape rates for 15 putative epitopes of which 14 elicited a response from patient T cells in ELISpot assays or are known epitopes or motifs for the patient HLA type [12, 13], supporting the presence of CTL-mediated selection. The sole exception, ENV830 in CH58, was lost by day 85 post symptoms, mutated through four different haplotypes during days *t*_1_, *t*_2_ and eight different haplotypes as infection progressed, dynamics suggestive of epitope shattering [30]. Columns *lower bound*, *intermediate* and *upper bound* correspond to the choices for *A*, *t*_*I*_ and *μ* described above, with the lower and upper bound representing a range in which we believe the escape rate lies, and the intermediate estimate describing a middle ground in which the parameters are not chosen to be extreme. The column *single* gives the escape rate estimate produced through Eq (1), which considers escape at each epitope separately. The column *previous* gives previous escape rate estimates from [13, 20] based on sample times up to 6 months and later, significantly beyond the 2–3 month range we consider.

**Table 5:** Novel method provides higher estimates of the escape rates for most of early escapes in 4 HIV-infected patients. We use experimental data on kinetics of HIV escape from multiple CTL responses (see Fig 1) and estimate escape rates using Eq (3) with parameters given in the ranges in Table 4. Column “single epitope” denotes estimate of the escape rate assuming independent escape using Eq (1). Column “previous” denotes values of escape rates as estimated in previous publications [13, 20]. All putative epitopes are supported by ELISpot assays or HLA association except for ENV830 in CH58. All estimates of the escape rates are given in day^−1^ units.

patient	epitopes	lower bound	intermediate	upper bound	single epitope	previous
CH40	POL80	0.08	0.17	0.27	-0.01	0.02
CH40	VIF57	0.03	0.11	0.2	0.09	0.03
CH58	ENV830	0.11	0.22	0.34	0.17	0.12
CH58	GAG240	0.07	0.17	0.28	0.08	0.08
CH58	NEF105	0.05	0.15	0.26	0.09	0.07
CH77	ENV350	0.28	0.77	3.22	0.21	0.36
CH77	NEF17	0.11	0.49	2.34	0.04	0.30
CH77	VPU57	0.15	0.55	2.53	0.01	0.05
CH77	NEF73	0.22	0.66	2.87	0.06	0.29
CH77	ENV605	0.22	0.66	2.87	0.01	0.01
CH256	VIF169	0.02	0.08	0.14	0	0.04
CH256	NEF185	0.06	0.13	0.19	0	0.08
CH256	ENV799	0.03	0.09	0.15	0.07	0.03
CH256	POL393	0.03	0.09	0.15	-0.02	0.03
CH256	ENV606	0.03	0.1	0.16	0.02	0.03

For CH58 and CH77, the lower bounds demonstrate that escape can proceed concurrently, with rates exceeding 0.05 day^−1^ at multiple epitopes. The lower bounds are less informative for CH40 and CH256. For both these patients, a single epitope has a lower bound escape rate exceeding 0.05 day^−1^, but the other bounds are lower, roughly 0.03 day^−1^.

Across all patients, the upper bound escape rates are significant, allowing for the possibility of fast escape at multiple epitopes. However, with the exception of patient CH77, the upper bounds do not exceed 0.4 day^−1^, roughly the upper range seen in previous studies that considered escape separately at each epitope [12, 20, 28], suggesting a general limitation in the escape rate that applies across multiple epitopes.

The large difference between our lower and upper bounds reflects a large range of dynamics consistent with our patient datasets. Therefore, current acute HIV infection datasets based on SGA sampling and sparse temporal sampling cannot accurately estimate escape rates without significant modeling assumptions. Our intermediate estimates provide one case of such modeling assumptions. The estimates, formed by typical values for *A*, *μ* and *t*_*I*_ seen in simulation, demonstrate that significant escape rates of roughly 0.1–0.2 day^−1^ across multiple epitopes are consistent with our data and reflects reasonable assumptions.

The single escape rate estimates tend to fall near our lower bound estimates, possibly reflecting a downward bias of Eq (1), as seen in our numerical studies (see Table 1). In some cases, the single escape rates are negative and so almost certainly reflect such downward bias. Previous estimates made in [13, 20] also tend to fall near our lower bounds and in some cases far below the lower bounds. For example, escape ENV605 in CH77 was predicted to occur at a rate 0.01 day^−1^ and our novel estimates suggest at least an escape rate of 0.22 day^−1^, a 22-fold increase. Since single estimates of escape rates are based on samples over a longer time period, their downward bias relative to our estimates suggests that escape rates may indeed slow down as time progresses as has been suggested previously [18, 20].

Overall, our analysis strongly suggests that escape in acute HIV infection occurs concurrently from multiple CTL responses and proceeds at significant rates. However, whether escape rates reach the ranges of 0.1–0.2 day^−1^ suggested by our intermediate values cannot be determined without more assumptions (leading to narrower rate for parameters *A*, *t*_*I*_, and *μ*) or denser temporal sampling.

## Discussion

We have presented a novel method for estimating the rate of concurrent escape of HIV from multiple CTL responses that can be applied to our dataset and potentially other datasets. The method is based on an escape graph representing the mutation pathways through which HIV evades CTL response. Our method extends the logistic model of [19, 21] to an escape graph by considering pairs of viral variants corresponding to parent-child vertices on the graph. Through stochastic simulations we have shown that the logistic method can be biased in complex ways by concurrent multi-epitope escape and severely downward biased when escape is captured at a single time point.

Our results suggest that CTL escape can occur concurrently at multiple epitopes, with escape rates ranging between 0.03 and 0.4 day^−1^ across multiple epitopes. The upper bound of 0.4 day^−1^ is in-line with upper bounds for CTL escapes found using the logistic model [12, 14, 15], suggesting a general limit for the rate of escape whether escape is proceeding concurrently at multiple epitopes or not. Our lower bounds are less informative but are greater than estimates of the escape rates based on methods that do not account for concurrent multi-epitope escapes. If replicative fitness costs are associated with epitope mutations, then CTL kill rates are higher than the escape rate estimates. But this effect would apply to previous estimates as well, so the comparison between our escape rate ranges and previous ranges still has validity. In a similar vein, we have grouped mutations at each epitope, meaning that our escape rate estimates are averages over the different mutation variants at a given epitope. As a result, our escape rate estimates will underestimate the escape rate of some mutation variants and overestimate others.

Pandit and De Boer [27] recently demonstrated the presence of concurrent CTL escape in a one patient dataset. Our results provide further confirmation of concurrent CTL escape through four patient datasets. However, the nature of CTL escape may differ between patients and differing conclusions may reflect the use of different datasets. Results by Kessinger et. al. [25] and da Silva [34] suggest non-concurrent escape, meaning that escape occurs one epitope at a time.

Kessinger et. al. [25] present simulations showing that escape is largely non-concurrent, but that (see their Fig 2) concurrent escape can occur, although with one variant at high frequency. Combining our results and those of Pandit and De Boer [27] with the analysis of Kessinger et. al. [25] suggests escape dynamics in which concurrent escape occurs and is resolved in favor of a given escape variant, which then serves as the basis of another concurrent escape that is resolved in favor of another escape variant, and so on. Further work will be required to verify this conjecture.

Da Silva calculated an effective population size (*N*_*e*_) for HIV of 10^2^–10^3^ during early infection based on a census population on the order of 10^7^. In simulations of CTL escape based on a Wright-Fisher model with *N*_*e*_ of 10^2^–10^3^, da Silva observed that escapes do not happen concurrently. Since our simulations assume census population sizes similar to da Silva’s (i.e. 10^7^), combining our results with those of da Silva raises the possibility that a Wright-Fisher model does not accurately reflect HIV escape dynamics, as has been suggested by previous authors [35, 36] (see also [37] for a non-HIV perspective).

Concurrent viral escape variants may affect each other indirectly through competition for target cells. Previous authors have considered such interactions and the potential role of clonal interference on viral evolution, see the reviews [38, 39] for further details. However, the role of target cell limitation in early HIV infection is still unclear [40–42]. Importantly, the specific dynamics of interaction between CTL escapes affect our escape rate estimates only through *A*, *t*_*I*_ and *μ*.

Our approach and results come with the statistical caveat that a biased escape graph will produce biased estimates. For example, the escape graph will be biased when many low frequency variants are present. To see why, consider an extreme example of CTL escape at 100 epitopes and imagine that 100 variants exist in the viral population, each variant mutated at a single epitope and each variant at frequency 1%. If we form an escape graph based on sampling 10 sequences, the most likely outcome is 10 different variants, each with sample frequency 10% but with true frequency 1%. Dataset CH77 has a sampling pattern consistent with such biasing. In general, formation of the escape graph is statistically complex and other forms of bias may exist, although such biases did not arise in our simulations. Exploration of this issue requires further work.

Much of our approach follows from the sparse sampling of current datasets. While the rise of deep sequencing datasets addresses the shallowness of sampling, understanding the complexity of early escape requires linkage information and would benefit from more sampling time points. Our methods and results highlight the importance of better sampled datasets for understanding early HIV dynamics and evolution.

## Methods

### Escape Graphs and Recombination

**Fig 3:**
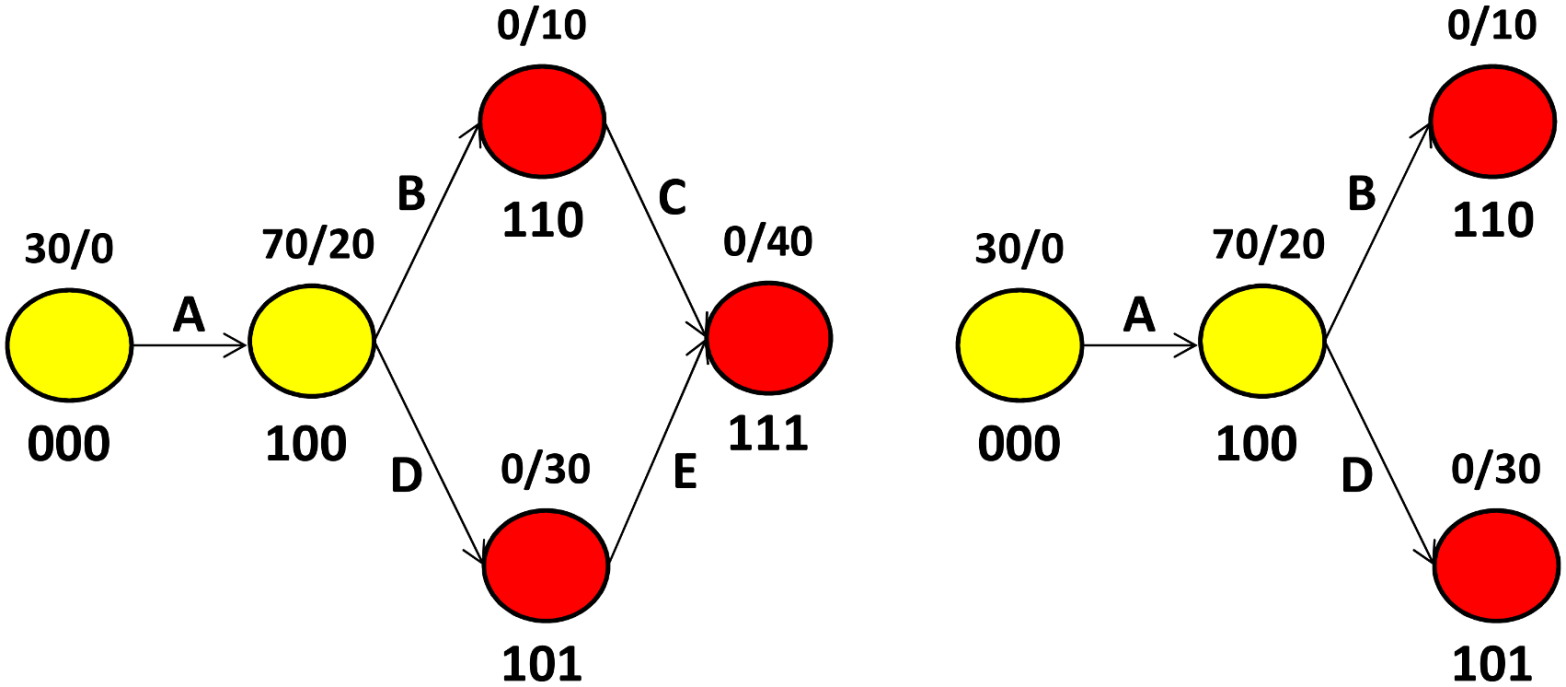
Two escape graphs generated using our stochastic simulation model. See text for details of simulations and Fig 1 for details of escape graph notation.

The left escape graph of Fig 3 shows escape at three epitopes. Multiple paths can be taken to reach variant 111 and recombination may play a role. Our current method does not account for recombination, but in many cases we can nevertheless obtain escape rate estimates.

Our goal in estimating escape rates is to quantify the CTL killing rates associated with CTL response at different epitopes. When multiple paths exist between two vertices, multiple edges must correspond to escape at the same epitope: here edges *B* and *E* correspond to escape in epitope 2, while edges *C* and *D* correspond to escape in epitope 3. In such cases, we specify a subgraph that includes a single escape rate for each epitope and we refer to such subgraphs as escape trees since each vertex possesses a single parent. The right graph of Fig 3 shows such an escape tree that is a subgraph of the escape graph on the left. The methods for estimating escape rates discussed above apply to escape trees, but issues of recombination associated with multiple paths are eliminated.

Ideally, we would estimates escape rates at all edges and this would provide information about CTL response in the context of different haplotypes. For example, in Fig 3, edges B and E correspond to escape at the same epitope, but the escape involves different haplotypes. Escape rates associated with the two edges need not be equal and estimating both might provide valuable information addressing the additivity of CTL killing across multiple epitopes. However, analyzing escape graphs with multiple paths would require more parameters to model recombination, an extension we have not explored.

In our datasets, the escape graphs of patients CH40 and CH77 require no pruning to reduce them to escape trees. The escape graph of patient CH58 requires the removal of two vertices, but the associated variants are at very low frequency at *t*_1_, 0.02 and 0.04 respectively, and are not sampled at *t*_2_. In CH256 we remove three vertices to produce an escape tree, with all three corresponding variants unsampled at *t*_1_ and sampled at 0.04 frequency at *t*_2_, see S1–S4 Figs for details.

### Stochastic Simulations

To perform simulations of viral escape, we use the model of Batorsky et. al. [32], developed for modeling chronic HIV infection, as a basis for a model of acute infection. The Batorsky et. al. model tracks individual viral genomes over time. Viral genomes, which correspond to provirus in infected cells, produce offspring, mutate and recombine. Selection is also modeled based on genome haplotype at *L* loci. As in the Batorsky et. al. model, we model selection at *L* loci, representing the CTL epitopes, and we account for the effects of mutation and recombination. In addition, we allow the number of viral genomes to vary, to account for viral expansion and contraction before and after peak viral load. Our model also includes time-varying selection as a model of CTL response. For simplicity, we assume all viral variants have equal replicative fitness.

For each simulation we track the CTL kill rate at a given epitope, providing us with the true escape rate (i.e. ϵ* in the notation of Eq (4)). Given a simulation, we then simulate sampling 15 sequences at *t*_1_ = 30 and *t*_2_ = 60, form an escape graph based on the simulated sequences, and then estimate the escape rate, *ϵ*, based on the inference method we are considering and the escape graph. Combining the true and estimated escape rates provides us with the relative error shown in the Tables 1–4. We produced 1,000 simulated escape graphs for each combination of strong and weak CTL response models and full and linear escape graphs (see caption of Table 1 for definitions) using a tau-leaping approach [43]. Table 6 gives the simulation parameters and their values. See S5 and S6 Figs for an example of a single simulation and the corresponding escape graph generated.

**Table 6:** Stochastic simulation parameters. *L*, *T*_on_, *k*_max,*i*_, *S*_*i*_, *α*, and *β* are parameters associated with CTL response. Values shown in the Dominant (Subdominant) column apply to response at the first (second-sixth) epitopes. *U* is a uniform random number on [0, 1], meaning that the timing and strength of the subdominant responses is different for each simulation. Values of *k*_max,*i*_ shown are for the strong CTL response simulation. Weak CTL response simulation differ only in *k*_max,*i*_ = .12*U* for the subdominant responses. *ρ*, *n*_rec_, and *μ* are parameters associated with recombination and mutation. CTL response parameters are chosen to qualitatively match responses seen in CTL datasets [11, 13] and match estimates given in [44].

parameter	meaning (units)	Dominant	Subdominant
*L*	number of epitopes	1	5
*T*_on_	time response initiates (day)	14	20 + 10 × *U*
*k*_max,*i*_	maximum kill rate (day^−1^)	0.4	0.3 × *U*
*S*_*i*_	saturation constant (log10(infected cells))	3	3
*α*	proliferation rate (day^−1^)	2	1.2
*β*	contraction rate (day^−1^)	0.4	0.4
*ρ*	recombination rate per nucleotide (day^−1^)	1.4/5 × 10^−5^	
*n*_rec_	breakpoints per recombination	5	
*μ*	mutation rate per epitope (day^−1^)	10^−4^	

To model CTL response at *L* = 6 epitopes—roughly matching the number seen in our patient datasets—we use a CTL dynamics model introduced by De Boer et. al. in [44, 45]. Letting *k*_*i*_(*t*) be the CTL kill rate at time *t* of a variant possessing epitope *i*, we set *k*_*i*_(*t*) = 0 for *t* ≤ *T*_on,*i*_, where *T*_on,*i*_ represents a time at which CTL response to epitope *i* initiates. For *t* > *T*_on,*i*_, *k*_*i*_(*t*) varies according to

$${\dot{k}}_{i}=f\left(N_{i}\right)\alpha k_{i}\left(1-\frac{k_{i}}{k_{\max,i}}\right)-\left(1-f\left(N_{i}\right)\right)\beta k_{i},$$ (7)

with *k*_*i*_(*T*_on,*i*_) = 0.01 giving the initial condition of the response. (See Table 6 for meaning of parameters.) De Boer et. al. modeled different types of CTLs and tracked the CTL population size (see equations 4–7 in [45]), but here we group all CTLs and assume that the CTL population varies proportionally with the CTL kill rate, allowing us to track *k*_*i*_(*t*). In our simulations, the CTL response to epitope 1 occurs early and is relatively strong compared to responses to the other epitopes, which expand later. Strong and weak CTL simulations differ based on the value of *k*_max,*i*_. Rates of expansion and contraction for the immune response (*α* and *β*) are taken from parameters for the CD8 T cell response to a virus in mice [44, 45]. These values appear to be much higher than rates of T cell expansion and contraction in humans following vaccination [46]. In our simulations, higher values of these parameters allow for rapid change in CTL killing efficacy. The impact of the slower dynamics on the kinetics of escape will be investigated elsewhere.

At time 0, the population starts with 1 variant possessing all 6 epitopes. The population size *N*—not to be confused with effective population size—is expanded from 1 to 10^7^ within the first 21 days, following estimates of the total number of infected cells in the body, and then collapses to 10^4.5^ over the following two weeks and subsequently holds steady [47]. The reduction from 10^7^ to 10^4.5^ variants is larger than estimated in [47], but matches better the magnitude of viral load decline seen during acute infection [1]. Variants other than 000000 arise through mutation and recombination occurring at rates *μ* and *ρ*. All mutations occur independently at each epitope and to each variant according to a Poisson process run at rate *μ* = 10^−4^.

Recombination occurs at rate (*ρ*/5)*Nf*_*A*_ *f*_*B*_ with *ρ* = 1.4 × 10^−5^ and where *f*_*A*_, *f*_*B*_ are the frequencies of the recombining variants *A* and *B*. When recombination does occur, we assume 5 breakpoints uniformly selected over a 10000 nucleotide genome, matching the number of breakpoints measured through in-vitro studies [48–50]. Overall this gives a per nucleotide per day recombination rate of *ρ*, matching the rate measured in [51]. We assume all epitopes are 1000 base pairs apart. In the simulations, when a recombination between variants A and B occurs, we increased the resulting recombined variant population by 1 and decrease either the population of *A* or *B* variants by 1, where we choose to decrease either *A* or *B* with probability 1/2.

### Patient Datasets and Escape Graphs

For full details regarding patient datasets CH40, CH58, CH77, and CH256 see [12, 13] and references therein. Briefly the patients were identified during acute infection: viral load data suggests CH58 and CH256 were identified several days prior to peak viral load while CH40 and CH77 were identified several days after peak viral load. Blood samples were taken at various time points after the onset of symptoms and at each time point SGA was used to sequence the 5’ and 3’ ends of viral DNA, meaning that our data covers the whole genome but does not include linkage information between the 5’ end (GAG, POL) and 3’ end.

For patients CH40 and CH58, we apply our methods to the first two time points sampled after the onset of symptoms. Escape at CH77 was very fast and broad, encompassing 9 loci by the first timepoint sampled, day 14 post symptoms. To study this early escape, we assume that 10 days prior to symptoms, a time likely about 5 days prior to peak viral load, sampling would have been homogeneous for the founder variant. We then use 10 days prior to symptoms as our first timepoint and day 14 past symptoms as our second timepoint. For CH256, the first timepoint sampled was homogeneous for the founder variant and the second timepoint possessed variation at a single loci, so we use the second and third timepoints as our two sample times.

To choose lower bounds for *t*_*I*_, we estimate the patients’ peak viral load times and picked a time 5 days earlier. S3 Table summarizes the lower bound values for *t*_*I*_, *t*_1_, and *t*_2_ that we use for each patient dataset along with the number of samples available for the 5’ and 3’ end at each time point.

For CH77 all of the putative epitopes are on the 3’ end, so construction of the escape graph follows directly from the data. For the other three patients we construct a full escape graph by attaching 5’ edges onto the 3’ escape graphs. CH58 and CH256 each has only a single putative epitope on the 5’ end, meaning we form the full escape graph by adding a single edge to the 3’ escape graph. CH40 has putative epitopes evenly split between the 5’ and 3’ ends. Since we lack linkage information, the full escape graph represents a guess on our part. But we attach 5’ escapes to parts of the 3’ escape graphs near the root vertex, if the 5’ escapes are actually attached further away from the root our estimated escape rates would be higher. S1–S4 Figs show the 5’ and 3’ escape graphs, as well as the full escape graph we constructed.

## Supporting Information

S1 Text Supporting Information. (PDF)

S1 Fig CH40 escape graphs. (TIF)

S2 Fig CH58 escape graphs. (TIF)

S3 Fig CH77 escape graphs. (TIF)

S4 Fig CH256 escape graphs. (TIF)

S5 Fig An example of a single simulation performed using our stochastic mathematical model. Shown are CTL kill rate profiles targeting 6 viral epitopes (panel A), the epitope mutation frequencies (panel B), the variant frequencies (panel C), and the rate at which each variant population produces mutants (panel D). The kill rate for a given variant was the sum of the kill rates across all epitopes in the variant haplotype, meaning that we assumed additive killing across epitopes for which there was a ‘0’ in the variant label shown in the legend. Epitope mutation frequencies were computed by summing up the frequencies of all variants mutated at the given epitope. The simulation was run with *t*_1_ = 30 and *t*_2_ = 60. The census population size *N* was chosen to rise exponentially from 1 to 10^7^ over the first 3 weeks of infection, collapsed to 10^4.5^ over the next two weeks, and then hold steady. We assumed no fitness cost of the escape mutations in these simulations (i.e., same replicative fitness for all variants). In Panel D, the rate (day^−1^) at which 000000 variants mutates rises to roughly 1000, we plot on a more modest scale to make the other variant mutations rates visible. The sudden changes in slope seen in Panel D for variant 100000 at times 21 and 35 reflect the sudden change in the *N* profile at peak viral load (day 21) and the end of population collapse (day 35). This particular example assumes strong subdominant CTL responses that rise after the first CTL response.
(TIF)

S6 Fig Escape graphs from a single simulation. We simulated HIV evolution using a stochastic model as described in the Methods and graph the pathways of viral escape from 6 CTL responses. Panel A shows the escape graph generated by considering all variants with frequencies greater than 0.01 at either *t*_1_ or *t*_2_, and panel B shows the escape graph generated by random sampling of 15 sequences at times *t*_1_ and *t*_2_. For example, 2 of the 15 samples at *t*_1_ were viral variant 100000, which is a frequency of 13% as shown in the panel B. Edges in the escape graph give the epitope mutated in moving from parent to child. Initial and expansion variants are colored red and yellow, respectively.
(TIF)

S1 Table Putative epitopes supported by ELISpot assays presented in [12, 13] are highly enriched for mutation at times *t*_1_, *t*_2_. Shown are the total number of nucleotides sites spanned by all such epitopes and in parenthesis the percentage of the viral genome covered by such epitopes (epitope sites), the total number of variable sites within all such epitopes and in parenthesis the percentage of these variable sites relative to the number of variable sites across the viral genome (epitope variable sites), and the p-value assuming all sites across the genome are equally likely to be variable (p-value).
(PDF)

S2 Table Identical results as shown in Table 3, but here the CI are included. (PDF)

S3 Table For each patient lower and upper bounds for *t*_*I*_ and values of *t*_1_ and *t*_2_ used to form escape rate estimates are given. All times are in units of days since the onset of symptoms. 5’ samples and 3’ samples give the number of sequences sampled for each 1/2 genome at *t*_1_ and *t*_2_, respectively.
(PDF)
