# Supplementary material for: Broad CTL Response in Early HIV Infection Drives Multiple Concurrent CTL Escapes
Source: PLoS Comput Biol. 2015 Oct 27;11(10):e1004492. doi: 10.1371/journal.pcbi.1004492 (PMC4624722; doi:10.1371/journal.pcbi.1004492)
Supplement: S1 Text — (PDF) [file pcbi.1004492.s001.pdf]

Supporting Information

Patient Escape Graphs

S1 Fig-S4 Fig show the 5', 3', and full escape graphs for CH40, CH58, CH77 and CH256. Vertices are colored yellow and red to represent initial and expansion vertices. Uncolored vertices represent vertices we ignored in forming an escape tree as described in the Methods. The 5' epitopes are underlined in the full escape graph. Above each vertex is the associated variant frequency at the first and second sample times, respectively. Frequencies don't sum to 100 due to rounding.

S1 Figure

CH40 escape graphs.

S2 Figure

CH58 escape graphs.

S3 Figure

CH77 escape graphs.

S4 Figure

CH256 escape graphs.

Stochastic Simulations

S5 Fig and S6 Fig show a single simulation and the resultant escape graph. S5 Fig shows the profiles for the kill rates associated with $L = 6$ CTL responses, epitope frequency dynamics, variant frequency dynamics, and the rate at which each variant population produces mutants. The CTL kill rates rise and then fall as the associated epitope mutation frequencies rise (panels A and B), reflecting the effect of antigenic stimulation on CTL expansion and contraction. While epitope mutation frequencies rise monotonically, variant frequencies are more complex (panel C). The rise of a particular variant population, e.g. variant 100000, depends on the population size of its parent population, e.g. variant 000000. Panel D plots $\mu m_P(t)$ (see eqn (4) in main text for notation) for the different parent variants. When $\mu m_P(t) \approx 1$, the first child variant arises which is by definition the time t_I. We have $A = 1$ when mutations occur exactly at rate $\mu m_P(t)$ with stochastic deviations around this rate leading to deviations from $A = 1$.

Based on the simulated dynamics we sampled an escape graph, shown in S6 Fig B, at times $t_1 = 30$ and $t_2 = 60$ assuming 15 sampled sequences. S6 Fig A shows the escape graph including all variants with frequency $> .01$ at t_1 or t_2: the escape graph in S6 Fig B is a sample from the escape graph in S6 Fig A based on 15 sampled sequences. Escape rates were estimated using the frequencies given by the sampled escape graph of S6 Fig B since this sampled escape graph corresponds to what is available from our patient datasets.

S5 Figure

An example of a single simulation performed using our stochastic mathematical model. Shown are CTL kill rate profiles targeting 6 viral

epitopes (panel A), the epitope mutation frequencies (panel B), the variant frequencies (panel C), and the rate at which each variant population produces mutants (panel D). The kill rate for a given variant was the sum of the kill rates across all epitopes in the variant haplotype, meaning that we assumed additive killing across epitopes for which there was a '0' in the variant label shown in the legend. Epitope mutation frequencies were computed by summing up the frequencies of all variants mutated at the given epitope. The simulation was run with $t_1 = 30$ and $t_2 = 60$. The census population size N was chosen to rise exponentially from 1 to 10^7 over the first 3 weeks of infection, collapsed to $10^{4.5}$ over the next two weeks, and then hold steady. We assumed no fitness cost of the escape mutations in these simulations (i.e., same replicative fitness for all variants). In Panel D, the rate (day^{-1}) at which 000000 variants mutates rises to roughly 1000, we plot on a more modest scale to make the other variant mutations rates visible. The sudden changes in slope seen in Panel D for variant 100000 at times 21 and 35 reflect the sudden change in the N profile at peak viral load (day 21) and the end of population collapse (day 35). This particular example assumes strong subdominant CTL responses that rise after the first CTL response.

S6 Figure

Escape graphs from a single simulation. We simulated HIV evolution using a stochastic model as described in the Methods and graph the pathways of viral escape from 6 CTL responses. Panel A shows the escape graph generated by considering all variants with frequencies greater than 0.01 at either t_1 or t_2, and panel B shows the escape graph generated by random sampling of 15 sequences at times t_1 and t_2. For example, 2 of the 15 samples at t_1 were viral variant 100000, which is a frequency of 13% as shown in the panel B. Edges in the escape graph give the epitope mutated in moving from parent to child. Initial and expansion variants are colored red and yellow, respectively.

Evidence of CTL Mediated Selection

Of the putative epitopes we considered, those supported by ELISpot assays were highly enriched for variation in sampled sequences, providing statistical evidence of CTL mediated selection. As shown in S1 Table, such epitopes composed $3 - 11\%$ of the viral genome but contained $20 - 33\%$ of the variable sites over all the sequences collected during times t_1, t_2. A null model assuming a uniform distribution of variable sites across the genome is rejected with p-values shown in the table. The sequence widths of most putative epitopes were based on 18-mer peptides, making the percentage of nucleotides sites within epitopes an overestimate and, as a result, the p-values shown are also overestimates.

S1 Table

Putative epitopes supported by ELISpot assays presented in [12,13] are highly enriched for mutation at times t_1, t_2. Shown are the total number of nucleotides sites spanned by all such epitopes and in parenthesis the percentage of the viral genome covered by such epitopes (epitope sites), the total number of variable sites within all such epitopes and in parenthesis the percentage of these variable sites relative to the number of

variable sites across the viral genome (epitope variable sites), and the p-value assuming all sites across the genome are equally likely to be variable (p-value).

S2 Table

Identical results as shown in Table 3, but here the CI are included.

S3 Table

For each patient lower and upper bounds for t_I and values of t_1 and t_2 used to form escape rate estimates are given. All times are in units of days since the onset of symptoms. 5' samples and 3' samples give the number of sequences sampled for each 1/2 genome at t_1 and t_2, respectively.
