# Supplementary figures and images for: Broad CTL Response in Early HIV Infection Drives Multiple Concurrent CTL Escapes
Source: PLoS Comput Biol. 2015 Oct 27;11(10):e1004492. doi: 10.1371/journal.pcbi.1004492 (PMC4624722; doi:10.1371/journal.pcbi.1004492)

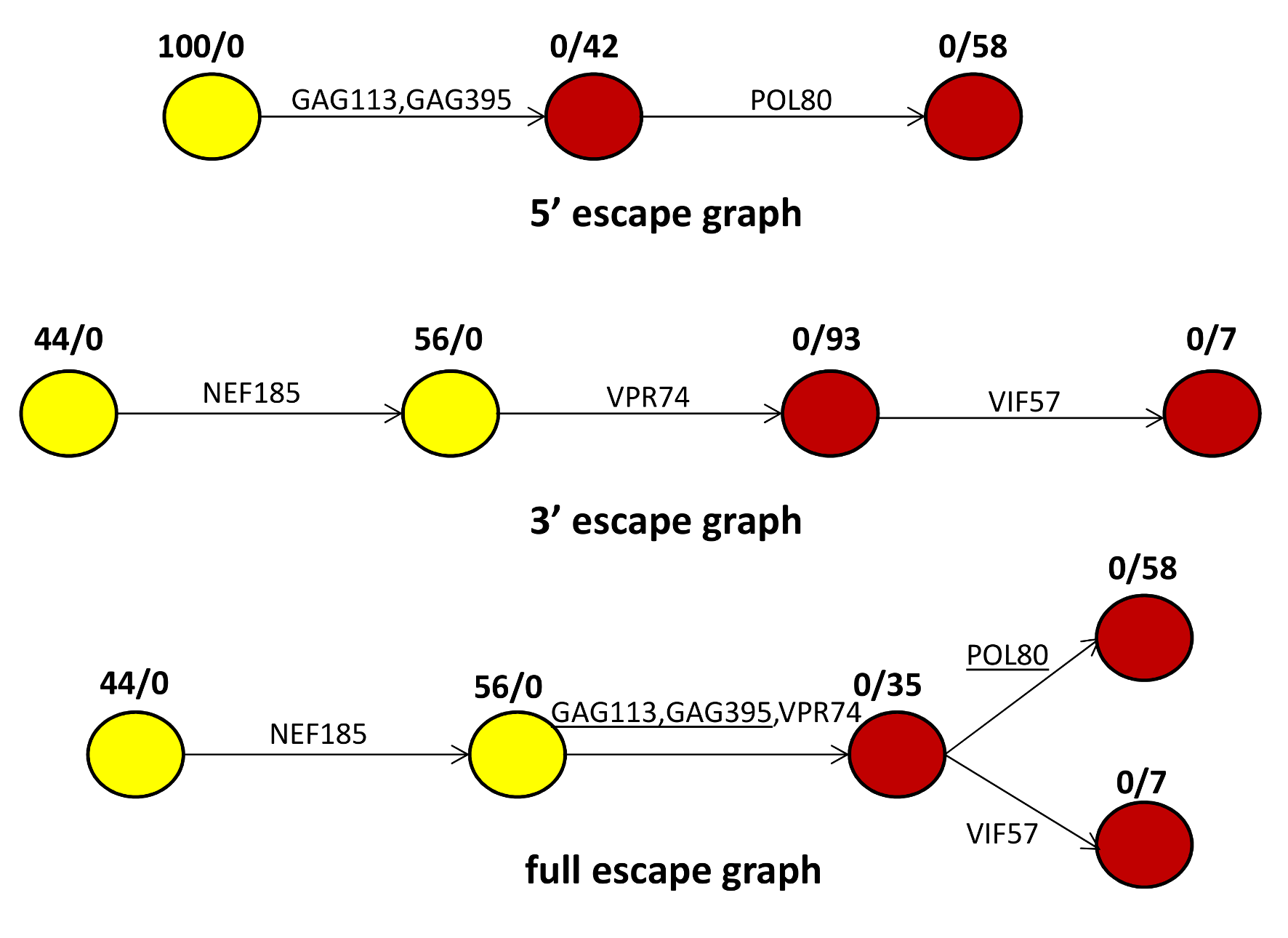

Supplement: S1 Fig — (TIF) [file pcbi.1004492.s002.tif]

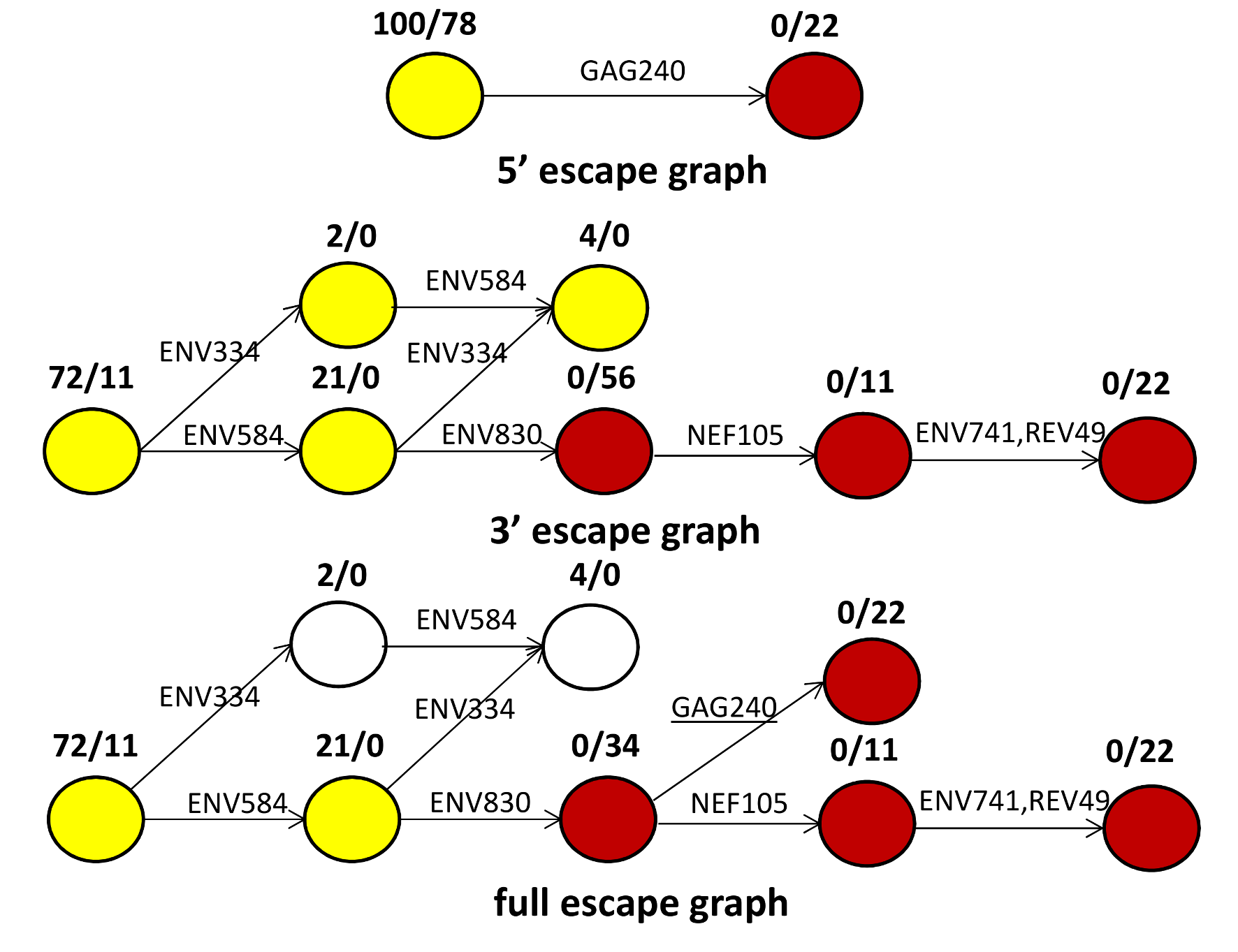

Supplement: S2 Fig — (TIF) [file pcbi.1004492.s003.tif]

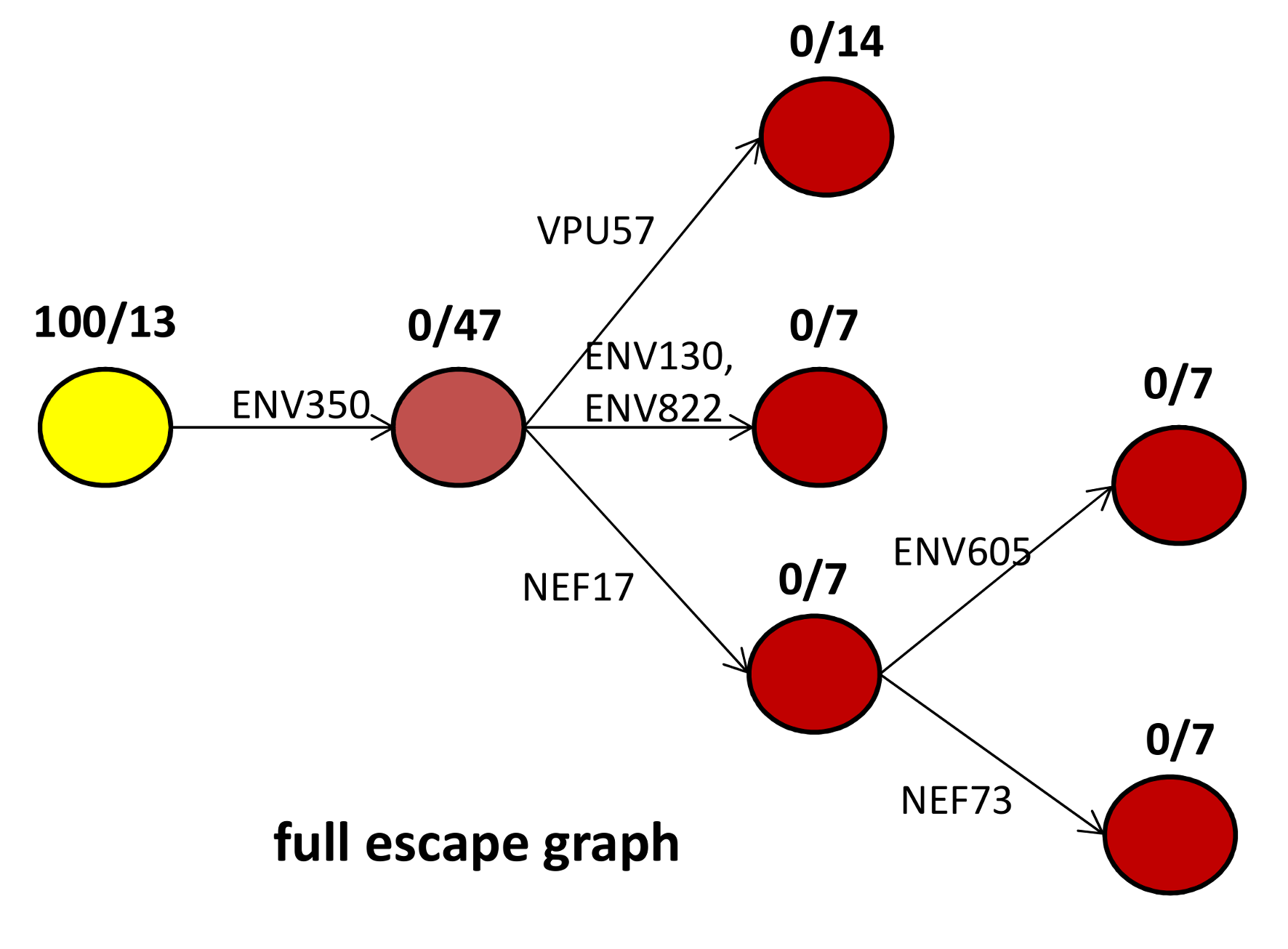

Supplement: S3 Fig — (TIF) [file pcbi.1004492.s004.tif]

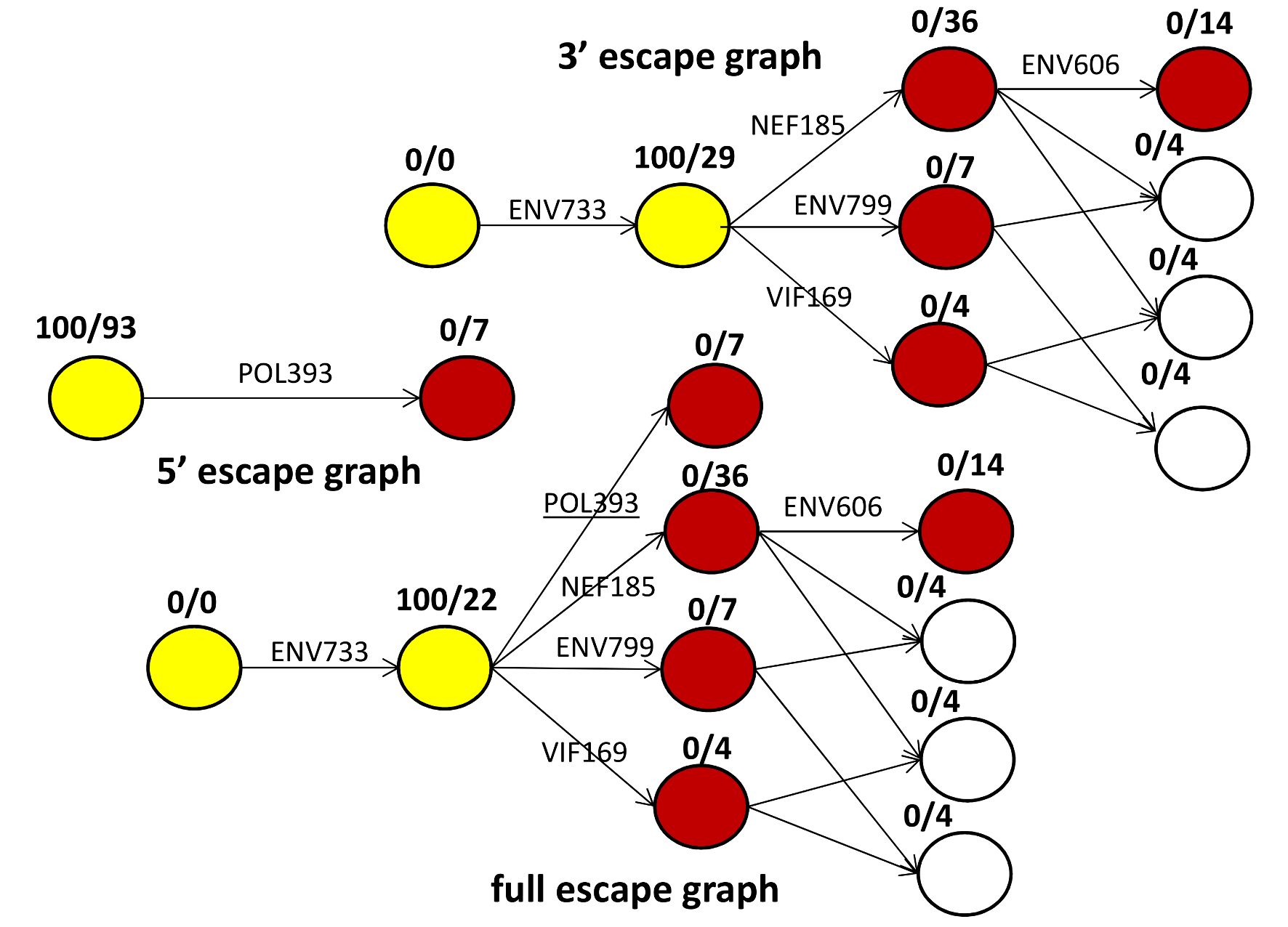

Supplement: S4 Fig — (TIF) [file pcbi.1004492.s005.tif]

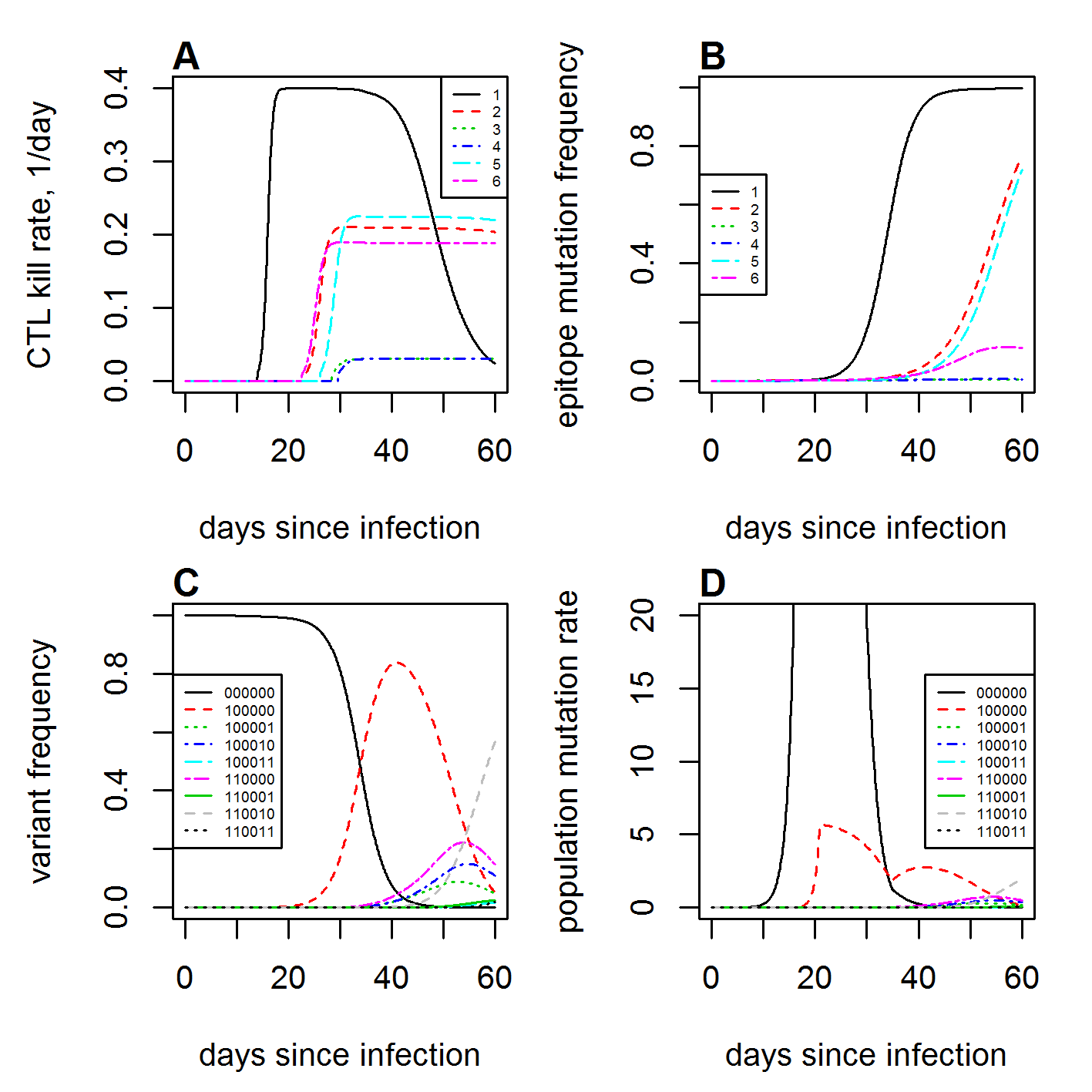

Supplement: S5 Fig — Shown are CTL kill rate profiles targeting 6 viral epitopes (panel A), the epitope mutation frequencies (panel B), the variant frequencies (panel C), and the rate at which each variant population produces mutants (panel D). The kill rate for a given variant was the sum of the kill rates across all epitopes in the variant haplotype, meaning that we assumed additive killing across epitopes for which there was a ‘0’ in the variant label shown in the legend. Epitope mutation frequencies were computed by summing up the frequencies of all variants mutated at the given epitope. The simulation was run with t 1 = 30 and t 2 = 60. The census population size N was chosen to rise exponentially from 1 to 107 over the first 3 weeks of infection, collapsed to 104.5 over the next two weeks, and then hold steady. We assumed no fitness cost of the escape mutations in these simulations (i.e., same replicative fitness for all variants). In Panel D, the rate (day−1) at which 000000 variants mutates rises to roughly 1000, we plot on a more modest scale to make the other variant mutations rates visible. The sudden changes in slope seen in Panel D for variant 100000 at times 21 and 35 reflect the sudden change in the N profile at peak viral load (day 21) and the end of population collapse (day 35). This particular example assumes strong subdominant CTL responses that rise after the first CTL response. (TIF) [file pcbi.1004492.s006.tif]

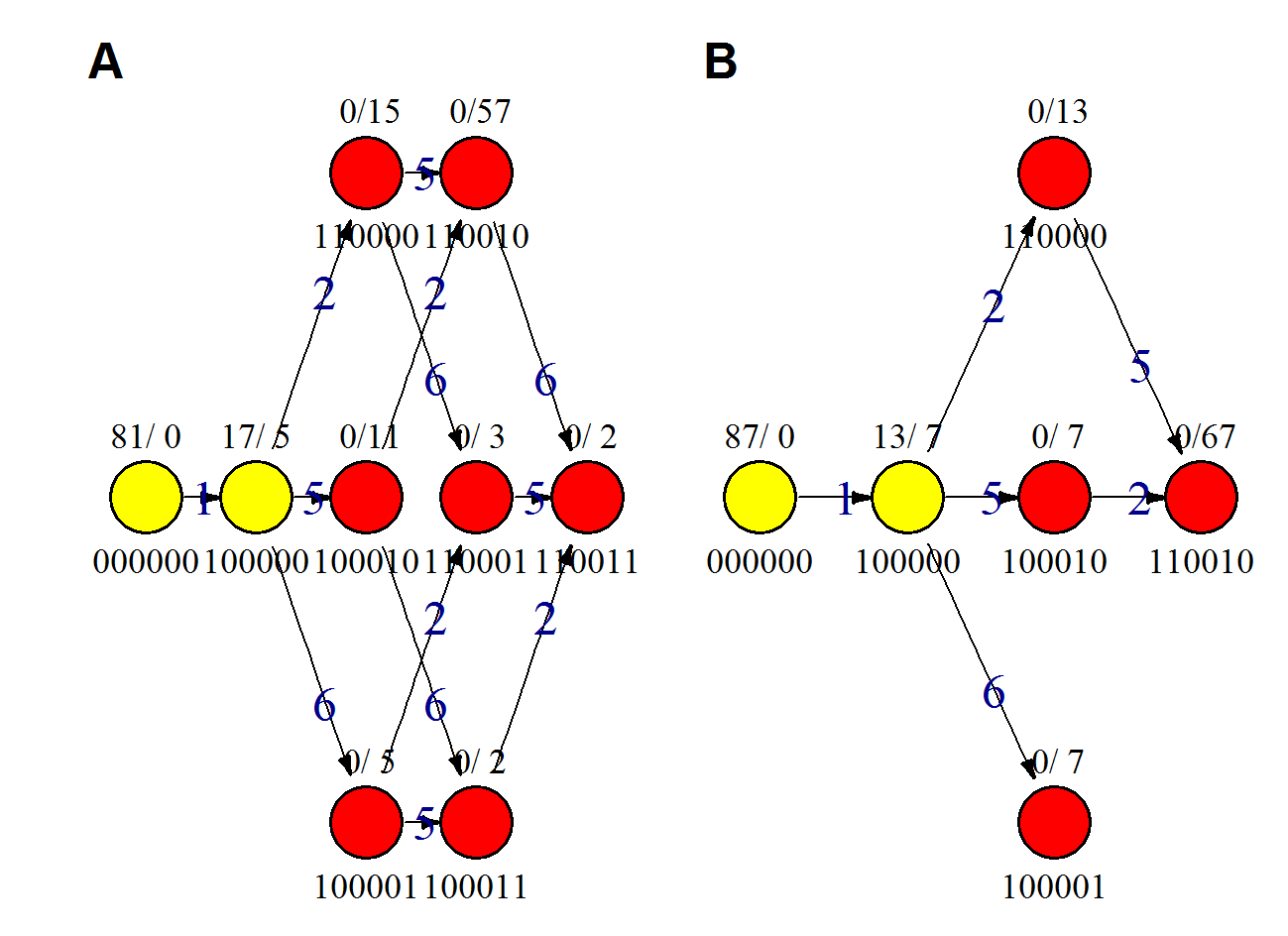

Supplement: S6 Fig — We simulated HIV evolution using a stochastic model as described in the Methods and graph the pathways of viral escape from 6 CTL responses. Panel A shows the escape graph generated by considering all variants with frequencies greater than 0.01 at either t 1 or t 2, and panel B shows the escape graph generated by random sampling of 15 sequences at times t 1 and t 2. For example, 2 of the 15 samples at t 1 were viral variant 100000, which is a frequency of 13% as shown in the panel B. Edges in the escape graph give the epitope mutated in moving from parent to child. Initial and expansion variants are colored red and yellow, respectively. (TIF) [file pcbi.1004492.s007.tif]
