# Supplementary material for: Broad CTL Response in Early HIV Infection Drives Multiple Concurrent CTL Escapes
Source: PLoS Comput Biol. 2015 Oct 27;11(10):e1004492. doi: 10.1371/journal.pcbi.1004492 (PMC4624722; doi:10.1371/journal.pcbi.1004492)
Supplement: S1 Table — Shown are the total number of nucleotides sites spanned by all such epitopes and in parenthesis the percentage of the viral genome covered by such epitopes (epitope sites), the total number of variable sites within all such epitopes and in parenthesis the percentage of these variable sites relative to the number of variable sites across the viral genome (epitope variable sites), and the p-value assuming all sites across the genome are equally likely to be variable (p-value). (PDF) [file pcbi.1004492.s008.pdf]

patient	epitope sites (%)	epitope variable sites (%)	p-value
CH40	618 (7%)	12 (22%)	3×10^{-4}
CH58	294 (3%)	18 (20%)	1×10^{-9}
CH77	1008 (11%)	14 (33%)	2×10^{-4}
CH256	690 (8%)	33 (23%)	1×10^{-8}
