# Supplementary material for: Broad CTL Response in Early HIV Infection Drives Multiple Concurrent CTL Escapes
Source: PLoS Comput Biol. 2015 Oct 27;11(10):e1004492. doi: 10.1371/journal.pcbi.1004492 (PMC4624722; doi:10.1371/journal.pcbi.1004492)
Supplement: S2 Table — (PDF) [file pcbi.1004492.s009.pdf]

CTL response	graph	$A = .75$	$A = 10$
strong	linear	0.07 (0.04,0.17)	-0.35 (-0.48,-0.27)
weak	linear	0.12 (0.08,0.21)	-0.82 (-0.91,-0.75)
strong	full	0.13 (0.05,0.42)	-0.28 (-0.37,-0.17)
weak	full	0.18 (0.12,0.32)	-0.74 (-0.85,-0.61)

CTL response	graph	$t_I = 0$	$t_I = 30$
strong	linear	-0.38 (-0.43,-0.35)	0.43 (0.38,0.47)
weak	linear	-0.43 (-0.49,-0.4)	0.56 (0.5,0.64)
strong	full	-0.38 (-0.6,-0.35)	0.39 (-0.08,0.45)
weak	full	-0.42 (-0.48,-0.38)	0.57 (0.51,0.68)

CTL response	graph	$\mu = 3 \times 10^{-5}$	$\mu = 6 \times 10^{-4}$
strong	linear	0.22 (0.16,0.32)	-0.33 (-0.47,-0.25)
weak	linear	0.48 (0.39,0.72)	-0.69 (-0.82,-0.62)
strong	full	0.2 (0.15,0.32)	-0.29 (-0.42,-0.23)
weak	full	0.44 (0.36,0.69)	-0.65 (-0.76,-0.57)
