# Supplementary material for: Broad CTL Response in Early HIV Infection Drives Multiple Concurrent CTL Escapes
Source: PLoS Comput Biol. 2015 Oct 27;11(10):e1004492. doi: 10.1371/journal.pcbi.1004492 (PMC4624722; doi:10.1371/journal.pcbi.1004492)
Supplement: S3 Table — All times are in units of days since the onset of symptoms. 5’ samples and 3’ samples give the number of sequences sampled for each 1/2 genome at t 1 and t 2, respectively. (PDF) [file pcbi.1004492.s010.pdf]

patient	t_I lower bound	t_I upper bound	t_1	t_2	5' samples	3' samples
CH40	-10	9	16	45	$13, 12$	$9, 14$
CH58	-5	14	8	45	$7, 9$	$53, 9$
CH77	-10	9	-10	14	$-, 17$	$-, 15$
CH256	-5	14	28	63	$10, 30$	$12, 28$
